# Interictal burden attributable to episodic headache: findings from the Eurolight project

**DOI:** 10.1186/s10194-016-0599-8

**Published:** 2016-02-16

**Authors:** Christian Lampl, Hallie Thomas, Lars Jacob Stovner, Cristina Tassorelli, Zaza Katsarava, Jose Miguel Laínez, Michel Lantéri-Minet, Daiva Rastenyte, Elena Ruiz de la Torre, Colette Andrée, Timothy J. Steiner

**Affiliations:** Headache Medical Center, Linz, Austria; Department of Neurogeriatric Medicine and Remobilisation, Hospital of the Sisters of Charity, Linz, Austria; Department of Neuroscience, Norwegian University of Science and Technology, Trondheim, Norway; Norwegian Advisory Unit on Headache, St Olavs University Hospital, Trondheim, Norway; Headache Science Centre, C Mondino National Neurological Institute, Pavia, Italy; Department of Brain and Behavioural Sciences, University of Pavia, Pavia, Italy; Department of Neurology, University of Duisberg-Essen, Essen, Germany; Department of Neurology, Evangelical Hospital Unna, Unna, Germany; Department of Neurology, Hospital Clinico Universitario, University of Valencia, Valencia, Spain; Departement d’Evaluation et Traitement de la Douleur, Centre Hospitalo-Universitaire de Nice, Nice, France; INSERM/UdA, U1107, Neuro-Dol, Clermont-Ferrand, France; Lithuanian University of Health Sciences, Kaunas, Lithuania; Asociacion Española de Pacientes con Cefalea (AEPAC), Valencia, Spain; Department of Pharmaceutical Sciences, University of Basel, Basel, Switzerland; Department of Population Health, Luxembourg Institute of Health, Strassen, Luxembourg; Division of Brain Sciences, Imperial College London, London, UK

**Keywords:** Headache, Migraine, Tension-type headache, Interictal burden, Public health, Europe, Eurolight project, Global Campaign against Headache

## Abstract

**Background:**

Most primary headaches are episodic, and most estimates of the heavy disability burden attributed to headache derive from epidemiological data focused on the episodic subtypes of migraine and tension-type headache (TTH). These disorders give rise directly but intermittently to symptom burden. Nevertheless, people with these disorders may not be symptom-free between attacks. We analysed the Eurolight dataset for interictal burden.

**Methods:**

Eurolight was a cross-sectional survey using modified cluster sampling from the adult population (18–65 years) in 10 countries of the European Union. We used data from nine. The questionnaire included headache-diagnostic questions based on ICHD-II and several question sets addressing impact, including interictal and cumulative burdens.

**Results:**

There were 6455 participants with headache (male 2444 [37.9 %]). Interictal symptoms were reported by 26.0 % of those with migraine and 18.9 % with TTH: interictal anxiety by 10.6 % with migraine and avoidance (lifestyle compromise) by 14.8 %, both much more common than in TTH (3.1 % [OR 3.8] and 4.7 % [OR 3.5] respectively). Mean time spent in the interictal state was 317 days/year for migraine, 331 days/year for TTH. Those who were “rarely” or “never” in control of their headaches (migraine 15.2 %, TTH 9.6 %) had significantly raised odds of interictal anxiety, avoidance and other interictal symptoms. Among those with migraine, interictal anxiety increased markedly with headache intensity and frequency, avoidance less so but still significantly. Lost productive time was associated with high ORs (up to 5.3) of anxiety and avoidance.

A third (32.9 %) with migraine and a quarter (26.7 %) with TTH (difference: *p* < 0.0001) were reluctant to tell others of their headaches. About 10 % with each disorder felt families and friends did not understand their headaches. Nearly 12 % with migraine reported their employers and colleagues did not.

Regarding cumulative burden, 11.8 % reported they had done less well in education because of headache, 5.9 % reported reduced earnings and 7.4 % that their careers had suffered.

**Conclusions:**

Interictal burden in those with episodic headache is common, more so in migraine than TTH. Some elements have the potential to be profoundly consequential. New methodology is needed to measure interictal burden if descriptions of headache burden are to be complete.

## Background

Headache disorders, especially tension-type headache (TTH) and migraine, are extremely common [1]. From a public-health perspective, and also from the viewpoint of affected people, they are also among the most disabling: migraine is the sixth highest cause in the world of years of healthy life lost to disability (YLDs), and headache disorders collectively are third, according to the Global Burden of Disease Study 2013 (GBD2013) [2, 3]. Various causes of headache occurring on ≥15 days every month affect 2–4 % of the world’s adult population [4]. Among these, medication-overuse headache (MOH) affects 1–2 % [5], and this disorder is itself among the top 20 causes (18^th^) of YLDs [2, 3]. Nevertheless, most primary headaches are episodic, and most of the disability burden attributed to headache in GBD2013 was based on epidemiological data focused on the episodic subtypes of migraine and TTH. These disorders give rise directly, but intermittently, to symptom burden: pain, often accompanied in the case of migraine by nausea, vomiting and photo- and/or phonophobia. All of these tend to cause debility, prostration and reduced functional ability, a secondary disability burden which is the principal cause of YLDs and consequential lost productivity.

This *ictal* burden is easily conceptualised; but it has long been recognised that people with episodic headache may not be entirely symptom-free between attacks [6, 7]. There are good reasons for this. Since headache attacks are unpleasant, people who experience them wish not to do so. Those in whom they occur frequently are very likely to worry about when the next may happen, and in some this can reach a level of anxiety. More commonly it may provoke avoidance behaviour, particularly among those with migraine who identify triggers and endeavour to eliminate them by lifestyle compromise. Sensible this may be, but too much lifestyle compromise may take the pleasure out of life. An example given by Stovner et al. was this: “Leisure activities may be cancelled or curtailed because of headache; when many have been cancelled, social events are likely not to be planned in the first place. Social life between attacks may simply cease” [7]. These are elements of *interictal* burden.

The health and wellbeing importance of interictal burden lies in its continuity. Whereas the ictal burden of episodic headache is typically present during only one or two days in every month, interictal burden can impose itself on all of the other days. This means two things. First, interictal burden ought not to be ignored: the burden of headache is very poorly described if it does not take interictal burden into account. Second, if interictal burden is overestimated, then multiplied by time, quantification of overall burden is likely to be greatly distorted. GBD2010 described distinct ictal and interictal health states associated with both migraine and TTH, and allocated disability weights (DWs) to each, but the interictal DWs and burden estimates arising from them were not reported [1], probably for this reason.

In fact there is little empirical knowledge about interictal burden. Furthermore, while it has been described [6–10], it has no accepted definition. A single Swedish study has made a population-based estimate of prevalence [11], but this described only the proportion of people with migraine (43 %) who recovered completely between attacks. There are no published studies that have estimated magnitude. We may assume that significant relationships exist between interictal burden and the behaviour, performance, productivity, family life and social activities of those affected, but no data exist to confirm these.

The Eurolight project, supported by the European Commission Public Health Executive Agency, was a partnership activity within the Global Campaign against Headache. Its main purpose was to gather knowledge of the impact of headache disorders of public-health importance across Europe. Its questionnaire included a number of question sets designed to capture elements of headache-attributed burden, among which were those likely to be experienced interictally. We analysed the Eurolight dataset accordingly, and report our findings here. Our working definition of interictal burden was: “Any loss of health or wellbeing attributable to a headache disorder reportedly experienced while headache-free.” It has multiple components [10]; those addressed by the Eurolight questionnaire included interictal anxiety, avoidance behaviour and non-headache symptoms; perceptions of poor headache control, stigma and social isolation; and the cumulative burdens engendered by disturbed education, lost career opportunities and damaged family life.

## Methods

The original Eurolight survey was conducted from November 2008 to August 2009. The full methodology is described elsewhere [12]; below we present brief details. The survey was of cross-sectional design and used modified cluster sampling in 10 countries (Austria, France, Germany, Ireland, Italy, Lithuania, Luxembourg, Netherlands, Spain and United Kingdom) representing >60 % of the adult population (18–65 years) of the European Union. The sampling methods, summarised in Table 1, varied between countries according to what was feasible [12]. Additional samples in Spain and Netherlands, and the only sample in Ireland, were recruited through patients’ organisations [12]. To avoid the inevitable biases inherent in these, they were not included in this analysis.

**Table 1:** Summary of data collection methods in each country

Country	Sample size (n)			Methods
	Denominator	Responders	Responders with headache	
Austria	up to 6000	646	454	Up to 10 consecutive patients aged 18–65 y visiting any of 400 general practitioners (GPs) and 200 neurologists for any reason on a pre-specified day. Questionnaires to be completed and returned later. One reminder after one month to non-responders.
France	2400	876	586	Consecutive patients aged 18–65 y attending any of a cooperative of 80 GPs on a pre-specified day. Questionnaires to be completed and returned immediately or later by post. One reminder by email after one week to non-responders.
Germany	3000	338	248	Random urban (50 %) and rural (50 %) samples aged 18–65 y from general population listings supplied by local municipal authority. Questionnaires distributed and returned by post. No reminders sent.
Italy	3500	500	374	Random urban (70 %) and rural (30 %) samples drawn from general population using listings supplied by Azienda Sanitaria Locale of Pavia, stratified with regard to gender, age (in range 18–65 y) and education. Questionnaires distributed and returned by post. No reminders sent.
Lithuania	1137	616	440	Sample drawn from Kaunas city and Kaunas region using Residents’ Register Service, reflecting age (in range 18–65 y) and gender composition of Lithuania and proportions living in rural (33 %) or urban (67 %) areas. Data collection face-to-face, conducted by medical students “cold-calling” door-to-door.
Luxembourg	6498	2023	1473	Sample aged 18–65 y, stratified for age, gender, region and nationality, drawn from general population via national social security registry (IGSS). Questionnaires distributed and returned by post. Reminders sent one month later to non-responders.
Netherlands	unknown	2414	1993	Survey conducted by market research company with access to population sample of 200,000, representative with regard to gender, age (in range 18–65 y), region and education. Questionnaire distributed by internet, to be completed on-line. Study stopped when >2000 received back.
Spain	1700	999	797	Random sample of employees of companies operating in national postal services in 10 areas of Spain, stratified to be representative of general working population with regard to gender, age (in range 18–65 y) and education. Ten occupational health physicians delivered and took return of questionnaires. One telephone reminder to non-responders.
United Kingdom	720	128	90	Modified population-based sampling through 12 GP practices in 11 areas (in UK, virtually all residents are registered with local GP). Questionnaire given to consecutive patients aged 18–65 y attending for any reason over a period of time, to be completed and returned immediately, or later by post.

In all countries, the survey used the same structured questionnaire [13], a derivative of the HARDSHIP questionnaire [10], translated into the local languages following *Lifting The Burden*’s translation protocol for lay documents [14]. Demographic questions were followed by screening questions for headache and, in those screening positively, by headache-diagnostic questions based on ICHD-II [15]. Participants with more than one headache type were asked to report only on the one they considered most bothersome. Diagnoses were made by computerized algorithm [10]. This first identified, and separated, participants reporting headache on ≥15 days/month (of whom additional questions enquired into medication use), then to the remainder applied ICHD-II criteria for migraine, TTH, probable migraine and probable TTH in that order. Thus a diagnosis of TTH trumped probable migraine [15]. In the analyses, migraine and probable migraine were considered together, as were TTH and probable TTH [7, 16]. Probable MOH (pMOH) was assumed to be the diagnosis when headache frequency was ≥15 days/month, duration was >4 h, the question “Do you usually take medication to treat your headaches” was answered “yes”, and frequency of acute medication use was ≥15 days/month when the medication was simple analgesics only or ≥10 days/month when it was any other (compound analgesics, opioids, triptans and/or ergots). A diagnosis of pMOH trumped all other diagnoses.

The questionnaire included several question sets addressing impact, including interictal and cumulative burdens (Table 2). In addition, it imported, as modules, the Headache-Attributed Lost Time (HALT) Index [17], which enabled correlations of interictal with ictal burden, and the Hospital Anxiety and Depression Scale (HADS) [18].

**Table 2:** Questions on interictal and cumulative burdens attributable to headache

Domain of enquiry	Question	Response options (optimum response first)
Symptoms in the interictal period (questions specifically about the last day when the respondent did *not* have a headache)	On that day, were you anxious or worried about your next headache episode?	no; yes
	On that day, was there anything you could not do or did not do because you wanted to avoid getting a headache?	no; yes
	On that day, did you feel completely free from all headache-related symptoms?	yes; no
Control of headaches	Taking into account everything you do to treat your headaches, do you feel you are in control of your headaches?	always; often; sometimes; rarely; never
Stigma and social isolation	Do you avoid telling people that you have headaches?	no; yes
	Do you feel that your family and friends understand and accept your headaches?	yes; no
	Do you feel that your employer and work colleagues understand and accept your headaches?	yes; no
Cumulative burdens	Have your headaches interfered with your education?	no;
	(multiple response options possible)	yes, I did less well;
		yes, I gave up early
	Do you believe your headaches have made you less successful in your career?	no;
	(multiple response options possible)	yes, I have done less well;
		yes, I have taken an easier job;
		yes, I have taken long-term sick leave;
		yes, I have retired early;
		yes, I am on a disability pension
	Have your headaches affected your family planning?	no;
		yes, I have had fewer children;
		yes, I have avoided having children
	During the last 3 months, have your headaches caused difficulties in your love life?	no; yes
	Have your headaches caused a relationship to break down?	no;
		yes, they have caused separation;
		yes, they have caused divorce

### Statistical analysis

Analyses were performed at the Norwegian University of Science and Technology using SPSS version 21 and Microsoft Excel version 14.0.7153.5000. In most cases these were of participants’ responses to the various questions, summarised for all those with headache or by diagnosis (migraine, TTH or pMOH). We described categorical variables as proportions (n [%]) and continuous variables in terms of means and standard deviations (SDs). We assumed interictal burden was present on days without headache; therefore we calculated time spent in interictal state (in days/year) as [365 – (reported headache frequency in days/year)]. We reported this variable in terms of means and standard errors (SEs). We calculated odds ratio (ORs) and 95 % confidence intervals (CIs) to show associations in bivariate analyses. We used chi-squared and Fisher’s exact tests of significance.

### Ethics

The National Ethics Committee of Luxembourg gave overall approval of the protocol. Further approvals were obtained from national or local ethics committees wherever needed. Similarly, data protection approvals were obtained centrally in Luxembourg and at country levels in compliance with national and European laws.

In each country, prospective participants received a written information sheet explaining the project and enquiry, and their purpose.

## Results

### Demographics and diagnoses

In total there were 6455 participants with any headache from the nine countries (male 2444 [37.9 %], mean age 41.9 ± SD = 12.6 years; female 4011 [62.1 %], mean age 40.8 ± 12.1 years). Among these, 2959 were diagnosed with migraine, 3033 with TTH and 249 with pMOH (Table 3).

**Table 3:** Proportions of participants with headache reporting interictal burden, by headache type and gender

Headache type	Gender	N	Interictal anxiety	Interictal avoidance	Not free of all symptoms	Mean time in interictal state
			*n* (%) [95 % CI]	*n* (%) [95 % CI]	*n* (%) [95 % CI]	days/year (mean ± SE)
All	Female	4011	344 (8.6) [7.7–9.5]	441 (11.0) [10.0–12.0]	902 (22.5) [21.2–23.8]	316 ± 1
	Male	2444	157 (6.4) [5.4–7.4]	227 (9.3) [8.1–10.5]	575 (23.5) [21.8–25.2]	
	All	6455	501 (7.8) [7.1–8.5]	668 (10.3) [9.6–11.0]	1478 (22.9) [21.9–23.9]	
Migraine	Female	2042	224 (11.0) [9.6–12.4]	302 (14.8) [13.3–16.3]	522 (25.6) [23.7–27.5]	317 ± 1
	Male	917	91 (9.9) [8.0–11.8]	135 (14.7) [12.4–17.0]	248 (27.0) [24.1–29.9]	
	All	2959	315 (10.6) [9.5–11.7]	437 (14.8) [13.5–16.1]	770 (26.0) [24.4–27.6]	
Tension–type headache	Female	1657	51 (3.1) [2.3–3.9]	70 (4.2) [3.2–5.2]	283 (17.1) [15.3–18.9]	331 ± 1
	Male	1376	42 (3.1) [2.2–4.0]	72 (5.2) [4.0–6.4]	289 (21.0) [18.8–23.2]	
	All	3033	93 (3.1) [2.5–3.7]	142 (4.7) [3.9–5.5]	572 (18.9) [17.5–20.3]	
Probable medication–overuse headache	Female	188	53 (28.2) [21.8–34.6]	58 (30.9) [24.3–37.5]	76 (40.4) [33.4–47.4]	120 ± 4
	Male	61	19 (31.1) [19.5–42.7]	15 (24.6) [13.8–35.4]	25 (41.0) [28.7–53.3]	
	All	249	72 (28.9) [23.3–34.5]	73 (29.3) [23.6–35.0]	101 (40.6) [34.5–46.7]	

### Interictal anxiety, avoidance and other interictal symptoms

Table 3 shows the proportions responding adversely to each of the three questions (see Table 2), for all participants with headache and by diagnosis and gender. It also shows, by diagnosis, the time in days/year spent with interictal burden. With regard to the episodic headaches, about one quarter (26.0 %) of participants with migraine and just under one fifth (18.9 %) with TTH reported interictal symptoms, in each case rather more males than females. Interictal anxiety was reported by about 10 % of those with migraine, avoidance by about 15 %. Both were much more common in migraine than TTH (for interictal anxiety, OR 3.8 [95 % CI: 3.0–4.8]; for avoidance, OR 3.5 [95 % CI: 2.9–4.3]). All proportions were substantially higher in pMOH: we report them in Table 3 for comparative interest, but note that, in any headache occurring on ≥15 days/month, it is not easy to discern what is ictal and what is interictal. For this reason, we present no further analyses of pMOH.

About half (53.1 %) of those with migraine, and nearly three quarters (71.4 %) with TTH, were “always” or “often” in control of their headaches; on the other hand, 15.2 % with migraine and 9.6 % with TTH were “rarely” or “never” so (Table 4). Gender differences were notable in the proportions “always” in control, significantly favouring males: for migraine, chi-squared = 43.923, *p* < 0.0001; for TTH, chi-squared = 4.677, *p* = 0.0306.

**Table 4:** Proportions of participants with headache reporting degrees of control of their headaches, by headache type and gender

Headache type	Gender	N	In control of headaches				
			“Always”	“Often”	“Sometimes”	“Rarely”	“Never”
			*n* (%) [95 % CI]	*n* (%) [95 % CI]	*n* (%) [95 % CI]	*n* (%) [95 % CI]	*n* (%) [95 % CI]
Migraine	Female	2042	228 (11.2) [9.8–12.6]	812 (39.8) [37.7–41.9]	667 (32.7) [30.7–34.7]	214 (10.5) [9.2–11.8]	100 (4.9) [4.0–5.8]
	Male	917	187 (20.4) [17.8–23.0]	344 (37.5) [34.4–40.6]	243 (26.5) [23.6–29.4]	80 (8.7) [6.9–10.5]	56 (6.1) [4.6–7.7]
	All	2959	415 (14.0) [12.8–15.3]	1156 (39.1) [37.3–40.9]	910 (30.8) [29.1–32.5]	294 (9.9) [8.8–11.0]	157 (5.3) [4.5–6.1]
Tension–type headache	Female	1657	566 (34.2) [31.9–36.5]	608 (36.7) [34.4–39.0]	307 (18.5) [16.6–20.4]	87 (5.3) [4.2–6.4]	54 (3.3) [2.4–4.2]
	Male	1376	523 (38.0) [35.4–40.6]	470 (34.2) [31.7–36.7]	209 (15.2) [13.3–17.1]	79 (5.7) [4.5–6.9]	71 (5.2) [4.0–6.4]
	All	3033	1089 (35.9) [34.2–37.6]	1078 (35.5) [33.8–37.2]	516 (17.0) [15.7–18.3]	166 (5.5) [4.7–6.3]	125 (4.1) [3.4–4.8]

We enquired into whether the probability of reporting interictal burden would increase with greater reported ictal burden (Table 5), although we did this only among those with migraine because ictal burden generally remains low in TTH. Interictal anxiety increased markedly with headache intensity and frequency, avoidance less so but still significantly. This was not the case with interictal symptoms, where increased odds of reporting were significant only among those with very high attack frequencies (>90 days/year). Those reporting poor control of their headaches had significantly raised odds of interictal anxiety, avoidance and other interictal symptoms. Lost productive time measured by the HALT index, especially lost work time, was associated with quite high odds (OR up to 5.3) of reporting interictal anxiety and avoidance, although, with increasing lost work time beyond the range 12–22 days/3 months, these odds tended to decline. With lost household work, the opposite was the case (Table 5).

**Table 5:** Probability of reporting interictal burden according to measures of ictal burden in participants with migraine

Ictal burden measure		Probability of reporting interictal burden		
		Odds ratio [95 % CI]		
		Interictal anxiety	Interictal avoidance	Not free of all symptoms
Headache intensity (reference: “not bad”)	“bad”	2.8 [1.5–5.4]	1.6 [1.1–2.4]	1.1 [0.8–1.4]
	“very bad”	7.6 [4.0–14.7]	3.0 [2.0–4.6]	1.3 [1.0–1.8]
Headache frequency (days/year) (reference: ≤12)	13–24	2.4 [1.5–3.7]	1.6 [1.1–2.2]	1.1 [0.8–1.4]
	25–48	2.5 [1.7–3.8]	2.0 [1.5–2.8]	1.3 [1.0–1.6]
	49–90	3.7 [2.5–5.6]	2.7 [1.9–3.6]	1.3 [1.0–1.6]
	>90	6.4 [4.3–9.6]	2.5 [1.8–3.6]	1.8 [1.3–2.3]
In control of headaches (reference: “always”)	“often”	1.1 [0.7–1.9]	1.5 [1.0–2.2]	1.1 [1.1–1.9]
	“sometimes”	3.5 [2.1–5.6]	2.8 [1.9–4.1]	1.9 [1.4–2.5]
	“rarely” or “never”	4.1 [2.5–6.9]	2.6 [1.7–3.9]	2.5 [1.8–3.4]
Lost productive time (HALT index): lost work time (days/3 months) (reference: ≤11)	12–22	5.3 [3.6–7.9]	2.9 [1.9–4.3]	1.8 [1.2–2.6]
	23–33	4.2 [2.0–8.9]	2.3 [1.1–5.0]	1.8 [0.9–3.7]
	>33	4.2 [1.7–10.2]	2.7 [1.1–6.6]	1.9 [0.8–4.4]
lost household time (days/3 months) (reference: ≤11)	12–22	2.4 [1.7–3.6]	2.4 [1.7–3.4]	1.3 [0.9–1.8]
	23–33	3.6 [2.1–6.3]	2.3 [1.3–4.0]	1.8 [1.1–3.0]
	>33	5.3 [2.6–10.7]	3.8 [1.9–7.6]	1.7 [0.9–3.5]
lost work + household time + social events (days/3 months) (reference: ≤22)	23–44	4.1 [2.9–5.7]	2.9 [2.1–4.0]	1.5 [1.1–2.0]
	45–66	4.0 [2.2–7.2]	2.8 [1.6–5.0]	2.2 [1.3–3.6]
	>66	4.4 [2.3–8.3]	2.4 [1.3–4.5]	1.7 [1.0–3.1]

We wondered whether our enquiry into interictal anxiety might be influenced by general anxiety. We could test this, because our enquiry included HADS; this enabled us to compare, for prevalence of interictal anxiety, those with HADS-A scores in the normal range (<8), borderline cases (scoring 8–10) and those at or above the threshold score (11) for anxiety caseness [18]. We performed this analysis in those with migraine, in whom we found a clear gradient: 7.9 %, 10.4 % (OR 1.3 [95 % CI: 1.0–1.8]; *p* = 0.072) and 17.8 % (OR 2.3 [95 % CI: 1.7–3.0]; *p* < 0.0001) respectively. We also repeated the analysis shown in Table 3 after excluding participants with HADS-A scores ≥11 (*n* = 1166). All proportions with interictal symptoms were reduced, but most not by much: for example, in males with migraine, interictal anxiety was reduced from 9.9 to 7.0 % (Fisher’s exact: *p* = 0.0066), avoidance from 14.7 to 14.0 % (*p* = 0.6783); in females with migraine, interictal anxiety was reduced from 11.0 to 10.0 % (*p* = 0.3293), avoidance from 14.8 to 14.0 % (*p* = 0.5073). We did not undertake these analyses in participants with TTH because their level of interictal anxiety was so much lower, or in those with pMOH because of small numbers.

### Stigma and social isolation

A third of respondents (32.9 %) with migraine and a quarter (26.7 %) with TTH were reluctant to tell others of their headaches (Table 6). This difference was significant (chi-squared = 26.744; *p* < 0.0001). Gender differences were minor. About 10 % with each disorder felt their families and friends did not understand or accept their headaches. Nearly 12 % with migraine who were employed reported their employers and colleagues did not, but those with TTH apparently fared better (6.6 %; chi-squared = 37.962; *p* < 0.0001).

**Table 6:** Proportions of participants with headache responding adversely to questions on social isolation, by headache type and gender

Headache type	Gender	N	Avoid telling others	Family, friends don’t understand	N	Employer, colleagues don’t understand
			*n* (%) [95 % CI]	*n* (%) [95 % CI]		*n* (%) [95 % CI]
Migraine	Female	2042	691 (33.8) [31.8–35.9]	227 (11.1) [9.7–12.5]	1723	201 (11.7) [10.2–13.2]
	Male	917	282 (30.8) [27.8–33.8]	76 (8.3) [6.5–10.1]	753	85 (11.3) [9.0–13.6]
	All	2959	973 (32.9) [31.2–34.6]	303 (10.2) [9.1–11.3]	2476	292 (11.8) [10–5–13.1]
Tension–type headache	Female	1657	429 (25.9) [23.8–28.0]	146 (8.8) [7.4–10.2]	1298	74 (5.7) [4.4–7.0]
	Male	1376	382 (27.8) [25.4–30.2]	142 (10.3) [8.7–11.9]	1086	84 (7.7) [6.1–9.3]
	All	3033	811 (26.7) [25.1–28.3]	288 (9.5) [8.5–10.5]	2384	158 (6.6) [5.6–7.6]

### Cumulative burden

Finally we made enquiries among those with migraine about cumulative burdens. We did not conduct this analysis among those with TTH, which is less recognisably a lifelong condition. Overall, 11.8 % of participants with migraine reported that they had done less well in their education because of their headaches. The proportion was higher (14.1 %) in those aged <40 years. Reduced earnings were reported by 5.9 %, while 7.4 % believed their careers had suffered generally and they had done less well than they might, but for their headaches. A small proportion (2.1 %) had specifically taken easier jobs, and 1.4 % had taken long-term sick leave.

Difficulties in love life were reported by 17.6 %, with slightly more (18.8 %) among those under 40 years and a marked gender difference (males 12.8 %, females 19.7 %). About 1 % of respondents reported having fewer children, or had avoided having children altogether, because of their migraine. Very small proportions claimed that migraine had caused marital separation (0.5 %) or divorce (0.2 %).

## Discussion

This was a large study of well over 6000 participants drawn from nine countries of Europe. It suffered from the limitations of the Eurolight study: it was not entirely population-based, sampling methods differed between countries (although to some extent this was a strength, since it was not a purpose to compare countries [12]), and participation rates were very low in some [19]. Nevertheless, this analysis leaves little doubt that interictal burden exists as a significant consequence of episodic headache. It is more evident in migraine than in TTH, reflecting its strong relationship with ictal burden – which is demonstrated here in several ways – but it is an important finding that it is not restricted to migraine. Avoidance, effectively meaning some degree of daily lifestyle compromise, was reported by nearly 15 % (or one in seven) with migraine compared to just under 5 % (one in 21) with TTH. Worry about the next episode was expressed by just over 10 % of those with migraine and 3 % of those with TTH. These were differences by a factor of about 3; however, while one quarter (26.0 %) of participants with migraine reported that they were not entirely free of symptoms interictally, the proportion (18.9 %) with TTH who said the same was not so much lower. Our enquiry did not extend into what the nature of these other interictal symptoms might be: this is further work to be done.

Gender differences, incidentally, appeared to be minor. The main exception to this was in proportions “always” in control of their headaches, which favoured males with either migraine or TTH. The determinants of feeling in control are complex, and probably largely dependent on effectiveness of acute therapy, whereas the perception of not being in control is undoubtedly a contributor to interictal burden. Of those with migraine, 15 % were “rarely” or “never” in control, of those with TTH 10 %, without significant gender differences.

Time spent in the interictal state, during which these symptoms might continuously be present, was, on average, 317 days/year in those with migraine and 331 in those with TTH. There is a conceptual issue in the estimation of total interictal burden per year as the product: [(burden assessed for a single day) x (days/year spent in interictal state)]. This works for large groups, not for individuals. By enquiring into burden on the last day without headache, we assumed this was a random day for each participant. On any such random day, a representative proportion of participants would have experienced interictal symptoms. It is not implied that interictal symptoms are present throughout the time in the interictal state in every participant who reports such symptoms (although in some they may be), but rather that the size of the proportion of people affected remains more or less constant.

The randomness of this day might be questioned, since by definition it was the last day of the interictal period. Intuitively it might be expected that anxiety, at least, builds over time during this period. In fact the evidence is against this: the clear relationships between interictal symptoms and headache frequency (Table 5) indicate *inverse* relationships with length of interictal period.

The last day without headache was also the day before the last headache. There is a possibility, therefore, that this enquiry captured premonitory symptoms, particularly the non-specific third question (“On that day, did you feel completely free from all headache-related symptoms?”). This question had the highest proportion of adverse responses (Table 3), but was least associated with headache intensity or frequency (Table 5).

Time spent in the interictal state is, of course, inversely related to headache frequency. The relationship between overall interictal burden and headache frequency is likely to be complex; burden may be heavier with higher frequency, but it is not borne for so long. In migraine, there was a strong relationship between the probability of interictal anxiety and headache frequency, and a less strong relationship for avoidance.

Our enquiry into anxiety (or worry) was specifically directed towards the next headache episode, and not aimed at general anxiety (which is known to be comorbid with migraine and also related to attack frequency [9, 20–22]). Perhaps not unexpectedly, we found a clear association, in migraine, between interictal worry about the next attack and general anxiety: those meeting HADS caseness criteria for anxiety [18] were more than twice as likely to report interictal anxiety as those in the normal range. This association, which was graded, suggests an aetiological relationship, but says nothing about direction of causation. It should be noted that it was much less strong than many associations between interictal anxiety and measures of ictal burden (Table 5). Furthermore, when all participants meeting HADS caseness criteria for anxiety (18.1 % of the total) were removed from the analysis, proportions among the remainder with interictal symptoms (including interictal anxiety) were reduced but not greatly. In other words, general anxiety cannot account for more than a very small part of interictal anxiety.

The considerable reluctance to tell others of their headaches (one third [32.9 %] with migraine and a quarter [26.7 %] with TTH) may reflect natural reticence in discussing health issues more than stigma attaching specifically to headache. The difference between disorders was significant (*p* < 0.0001), whereas gender differences were minor. On the other hand, employers and colleagues appeared to be rather understanding of respondents’ headaches: 88–89 % in the case of migraine, and about 93 % in the case of TTH (which probably caused them less trouble). In fact, they were as accepting as families and friends (about 90 % with each disorder).

Our enquiries into cumulative burden acknowledged an additional dimension to interictal burden, potentially very important in the context of a lifelong disorder [6, 7]. Doing less well in education because of headache was reported by 11.8 % of participants with migraine. Admittedly this might be a subjective judgment based on recall, perhaps from long ago, but 11.8 % is a large and noteworthy proportion. We could not measure the consequences of disrupted education, but they could be huge over a lifetime, and this finding is a reminder, if it is needed, of the importance of headache in childhood and adolescence. Some of the consequences were, perhaps, evident in responses to subsequent questions: reduced earnings, reported by 5.9 %; careers suffering, reported by 7.4 %. Then there were those who had effectively given up: 2.1 % who had taken an easier job, and 1.4 % on long-term sick leave – small numbers, but these outcomes represented profound consequences for these people and their dependents.

Difficulties in love life were common (17.6 %), and here there was a marked gender difference (male-to-female ratio 2:3). The 1 % who had had fewer children because of their migraine reflected another profound life-affecting consequence.

There are few previous studies with which to make comparisons. Boardman et al. argued that migraine pain would lead to isolation from social, emotional and behavioural aspects of life, and could greatly impact on these between attacks, being in this regard similar to other painful syndromes [23]. A Swedish study among patients in a migraine clinic revealed less contentment and vitality and poorer sleep and sense of wellbeing, coupled with more subjective symptoms in the headache-free periods, than among a population-based control group [24]. A population-based study in the same country found that only 43 % of people with migraine recovered completely between attacks [11]. This implied a far higher proportion (57 %) not free of all symptoms interictally than we found (26 %), but there was no description of what type of symptoms persisted, and some participants might have had more or less chronic headache. In a UK study, at least two thirds (66 %) of 158 hospital employees with migraine reported some form of impact between attacks, including interference with family (54 %) or work (35 %) relationships, feelings of not being in control of their lives (34 %) and damaged chances of promotion (15 %) [25]. “Not in control of lives” cannot be compared with our enquiry “not in control of headaches”, but generally these findings indicate greater interictal impact than ours. However, participation rate in the survey was 45 %, there was gender bias (93 % of the 158 were female) and, taking the nature of the population also into account, interest bias was highly likely. In these and other reports [6–9], the existence of interictal burden has clearly been recognised; in the latter, furthermore, many of its elements have been well described [6–9]. This study, however, is the first to estimate their general population prevalence.

A limitation was that our questions mostly invited yes/no responses. Therefore we could capture prevalence but not magnitude. The methodology for magnitude estimation has yet to be developed: it does not exist in recommendations for burden estimation so far put forward [7]. What this study has shown is that the methodology is needed: the high prevalence of interictal burden demands it if the burden of headache is to be adequately described.

## Conclusions

Interictal burden in those with episodic headache is common, more so in migraine than in TTH, but, importantly, it is not restricted to those with migraine. It shows a relationship with ictal burden. Although we did not assess the magnitude of burden, some elements – in particular those that are cumulative – have the potential to be profoundly consequential. There is a need for new methodology to measure interictal burden if descriptions of headache burden are to be complete.
